# In Vivo Imaging of Trypanosome-Brain Interactions and Development of a Rapid Screening Test for Drugs against CNS Stage Trypanosomiasis

**DOI:** 10.1371/journal.pntd.0002384

**Published:** 2013-08-22

**Authors:** Elmarie Myburgh, Jonathan A. Coles, Ryan Ritchie, Peter G. E. Kennedy, Alex P. McLatchie, Jean Rodgers, Martin C. Taylor, Michael P. Barrett, James M. Brewer, Jeremy C. Mottram

**Affiliations:** 1 Wellcome Trust Centre for Molecular Parasitology, College of Medical, Veterinary and Life Sciences, University of Glasgow, Glasgow, United Kingdom; 2 Institute of Infection, Immunity and Inflammation, College of Medical, Veterinary and Life Sciences, University of Glasgow, Glasgow, United Kingdom; 3 Faculty of Infectious and Tropical Diseases, London School of Hygiene and Tropical Medicine, London, United Kingdom; New York University School of Medicine, United States of America

## Abstract

Human African trypanosomiasis (HAT) manifests in two stages of disease: firstly, haemolymphatic, and secondly, an encephalitic phase involving the central nervous system (CNS). New drugs to treat the second-stage disease are urgently needed, yet testing of novel drug candidates is a slow process because the established animal model relies on detecting parasitemia in the blood as late as 180 days after treatment. To expedite compound screening, we have modified the GVR35 strain of *Trypanosoma brucei brucei* to express luciferase, and have monitored parasite distribution in infected mice following treatment with trypanocidal compounds using serial, non-invasive, bioluminescence imaging. Parasites were detected in the brains of infected mice following treatment with diminazene, a drug which cures stage 1 but not stage 2 disease. Intravital multi-photon microscopy revealed that trypanosomes enter the brain meninges as early as day 5 post-infection but can be killed by diminazene, whereas those that cross the blood-brain barrier and enter the parenchyma by day 21 survived treatment and later caused bloodstream recrudescence. In contrast, all bioluminescent parasites were permanently eliminated by treatment with melarsoprol and DB829, compounds known to cure stage 2 disease. We show that this use of imaging reduces by two thirds the time taken to assess drug efficacy and provides a dual-modal imaging platform for monitoring trypanosome infection in different areas of the brain.

## Author Summary

*Trypanosoma brucei*, a parasite transmitted by the bite of tsetse fly, is responsible for the disease human African trypanosomiasis (HAT). In advanced stages of HAT, trypanosomes invade the central nervous system (CNS), resulting in an array of neurological symptoms, and eventually death. Existing drugs for treatment of HAT are highly unsatisfactory and new safe drugs are urgently needed. Currently, potential drugs for HAT are screened in a mouse model that relies on the emergence of trypanosomes from tissues and their detection in blood. This can take up to 200 days, making selection and further development of new drugs slow and costly. Here, we employ *in vivo* imaging and genetically modified trypanosomes to monitor parasite distribution throughout the body in live infected mice. Our bioluminescence imaging approach provides sensitive detection of trypanosomes at sites of infection, allowing more rapid and more effective *in vivo* screening of candidate HAT drugs. Higher resolution intra-vital microscopy was used to investigate trypanosome dynamics in the brain and their accessibility to drugs during infection. These approaches allow more sensitive real time tracking of trypanosomes during chronic infections and will provide new insights about trypanosome pathogenesis in future experiments.

## Introduction

Human African trypanosomiasis (HAT), also known as sleeping sickness, is endemic to sub-Saharan Africa [1], [2] and is almost always fatal if untreated. Although its prevalence fell to a position of near control in the 1960s, a breakdown of surveillance and treatment allowed its re-emergence, with an estimated 300,000 cases annually by 1998. Over the past decade there has again been a steady decline in disease incidence most likely due to increased surveillance, distribution of free drugs and implementation of several clinical trials, but previous experience suggests that eradication is by no means assured [3], [4].

Development of a vaccine for HAT is unlikely due to the process of antigenic variation [5], [6] and the control of the tsetse fly responsible for disease transmission is problematic [7]. Chemotherapy is thus fundamental to efforts to eliminate HAT [8]. However, current drugs for HAT are highly unsatisfactory; with varying degrees of toxicity, a need for costly parenteral administration, efficacy below 100% and resistance a growing problem [9], [10]. To address these issues, a “pipeline” of new compounds has now emerged, although only two compounds (fexinidazole and SCYX-7158) are in clinical trials with a third, CPD-0802 (DB829) in advanced preclinical trials [8]. However, these novel drugs may not fulfil the requirements of potency and pharmacokinetic and safety profiles needed to eradicate the disease.

A major confounding factor in the development of new drugs for HAT is the lack of an efficient model for the second stage of the disease, when trypanosomes have become manifest in the central nervous system (CNS). The current model involves infecting mice with the GVR35 strain of *Trypanosoma brucei brucei* which become established in the CNS by 21 days [11]. Candidate drugs are then given and possible recrudescence of infection monitored in blood samples taken over a period of 180 days. The time delay in obtaining results is clearly a hindrance to defining structure activity relationships in iterative rounds of chemical synthesis.

In recent years, the improved sensitivity of *in vivo* imaging has expanded its application to a broad range of basic biological questions including disease modeling and drug screening. In infection biology, successful models to screen for drugs and infection patterns have been established for a number of microbes including *Mycobacterium tuberculosis* [12], *Streptococcus pneumoniae* [13], *Leishmania* [14] and *Trypanosoma brucei* [15], [16]. To determine whether use of *in vivo* imaging could yield a shortened method for screening candidate stage two drugs, we generated transgenic strains of brain invasive, chronically infecting *T. brucei* that stably express firefly luciferase. We show here that bioluminescence imaging of infected mice can radically shorten the period required to assess the *in vivo* efficacy of candidate drugs for stage 2 trypanosomiasis.

## Materials and Methods

### Ethics statement

All animal experiments were performed in accordance with the Animals (Scientific Procedures) Act 1986 and the University of Glasgow care and maintenance guidelines. All animal protocols and procedures were approved by The Home Office of the UK government and the University of Glasgow Ethics Committee.

### Animal infections

Adult female CD-1 mice (20–30 g body weight) were purchased from Charles River Laboratories and maintained under specific pathogen-free conditions. Mice were infected with 5×10^4^ *Trypanosoma brucei brucei* 427 (WT, -LUC2 or –Rluc), or with 3×10^4^ *T. b. brucei* strain GVR35 (WT, -LUC2 or -mCherry) trypanosomes by intraperitoneal injection and monitored for parasitemia by counting trypanosomes, in blood taken from the tail vein, using a haemocytometer (sensitivity of 2×10^4^ parasites/ml).

### Generation of expression constructs

To generate the backbone construct (pTbAM) that would be used to integrate reporters into the ribosomal DNA (*rDNA*) loci, a 250 bp sequence corresponding to the *T. b. brucei rDNA* promoter was amplified from genomic DNA using a forward primer to introduce a *SacI* restriction site and a reverse primer that introduced *MluI* and *NotI* sites (Table S1). In addition, a 563 bp fragment of the *rDNA* non-transcribed spacer sequence was also amplified from genomic DNA using a forward and reverse primer that introduced *ApaI* and *KpnI* restriction sites. Both amplicons were sequentially digested with *SacI* and *NotI* or *ApaI* and *KpnI* respectively and then ligated either side of a puromycin resistance cassette flanked by *αβ Tubulin* intergenic regions into a pBluescript backbone to create pTb-R. An upstream 5′-untranslated region (UTR) gene regulatory element corresponding to the *T. b. brucei GPEET2* 5′UTR, was amplified using primers to introduce *NotI* and *XhoI* restriction sites. The *GPEET2* 5′UTR amplicon and pTb-R backbone were digested with *NotI* and *XhoI* and ligated to create pTbAM. The enhanced firefly luciferase gene, *luc2*, was amplified from pGL4.14 (Promega) using forward and reverse primers to introduce an *XhoI* site before and a *BamHI* site after the gene, and cloned into pGEMT. Following digestion with these restriction enzymes the *luc2* gene was cloned into the *XhoI*/*BamHI* digested pTbAM vector to create pTbR-LUC2 (pGL2116). The mCherry gene was amplified using primers to add *HindIII* and *BamHI* sites and cloned into pGEMT. *HindIII*/*BamHI* digested mCherry was cloned into pHD1034 (from C. Clayton, [17]) to generate pHD1034-mCherry (pGL2160), and into p2628 (from M. Carrington [18]) to generate pGL2036.

### Culture-adaptation of GVR35 WT

*T. brucei* GVR35 WT had previously only been passaged through mice and in order to generate a bioluminescent line the cells had to be adapted to *in vitro* culture. Mice were infected with the mouse-passaged GVR35 WT line and monitored for parasitemia daily. During the first peak of parasitemia while trypanosomes were still dividing, blood was harvested and added to flasks containing different types of media and a range of fetal calf serum concentrations at 37°C, 5% CO_2_. GVR35 trypanosomes grew only in IMDM (Iscove's Modified Dulbecco's Medium, Gibco) supplemented with 20% heat-inactivated fetal calf serum (PAA), 20% Serum Plus, 0.75 mM hypoxanthine in 0.1 N NaOH, 4.1 mM glucose, 0.12 mM thymidine, 1.5 mM sodium pyruvate, 0.037 mM bathocuproine disulphonic acid, 0.2 mM β-mercaptoethanol, 1.1 mM L-cysteine, 0.38 mM adenosine, 0.38 mM guanosine, 0.83 g.L^−1^ methylcellulose, 0.04 mM kanamycin, 75 units.ml^−1^ penicillin and 0.075 mg.ml^−1^ streptomycin (all Sigma-Aldrich). Trypanosomes were fully adapted to culture and growing at a constant rate of 3-fold overnight after a month of culture. At this stage stabilates were made in supplemented IMDM medium containing 10% glycerol and frozen in liquid nitrogen for future use. All genetic modifications were done on culture-adapted GVR35 WT cells within a week after defrosting stabilates. For both WT and transgenic GVR35, *in vitro* culturing was kept to a minimum to avoid effects on virulence.

### *T. brucei* culturing and transfections

Culture-adapted *Trypanosoma brucei brucei* strain GVR35 bloodstream forms were grown *in vitro* at 37°C, 5% CO_2_ in supplemented IMDM medium (as described in Culture-adaptation of GVR35 WT). For generation of bioluminescent GVR35 lines 20 µg of *KpnI*/*SacI*-linearized pTbR-LUC2 plasmid was transfected into 3×10^7^ mid-log GVR35 WT trypanosomes using the Human T-cell Solution and Amaxa Nucleofector (Lonza) set on program X-001. GVR35-mCherry lines were generated by transfection using *NotI*-linearized pHD1034-mCherry plasmid. After recovery for 24 hours transformed clones were selected by limiting dilution in the presence of 0.15 µg.ml^−1^ puromycin (Calbiochem). *T. brucei* Lister 427 bloodstream form cells were grown in HMI-9 medium supplemented with 20% heat-inactivated fetal calf serum (PAA), 50 units.ml^−1^ penicillin and 50 µg.ml^−1^ streptomycin (Sigma) at 37°C, 5% CO_2_. Reporter 427 lines were generated by transfecting 1×10^7^ mid-log 427 WT cells with 20 µg of linearized plasmid using the Human T-cell Solution and Amaxa Nucleofector (Lonza). *NotI*-digested pGL2036 plasmid was used to generate 427-mCherry while *NotI*-digested Rluc-pHD309 [15] and *KpnI*/*SacI*-digested TbR-LUC2 was used to generate bioluminescent lines. After recovery for 6 hours transformed clones were selected by limiting dilution in the presence of appropriate antibiotics 5 µg.ml^−1^ hygromycin B (Calbiochem) or 0.5 µg.ml^−1^ puromycin (Calbiochem).

### *In vitro* luciferase assay

Mid-log bloodstream form trypanosomes, grown *in vitro*, were centrifuged at 1500 *g* for 10 minutes. Cells were resuspended in 100 µl of RPMI and added to 100 µl reconstituted luciferase assay reagent (Promega). For the analysis of clones, bioluminescence from 1×10^6^ cells was measured at different times after addition of substrate using an EnVision plate reader (PerkinElmer), and expressed as relative light units. For the *in vitro* detection limit assay, bioluminescence from 10–10^7^ cells was measured in 96-well plates using an IVIS Spectrum (Caliper Life Sciences) and expressed as total flux in photons per second.

### *In vitro* drug sensitivity assays

The IC_50_ values for trypanocides were determined by using a modified Alamar Blue assay. Cells were grown in IMDM medium supplemented as described earlier and assays performed in duplicate on three independent occasions. Briefly, mid-log strain GVR35 WT and GVR35-LUC2 trypanosomes were added to a dilution series of drugs in IMDM at a final density of 3×10^4^ cells/ml. After incubation for 48 hours at 37°C, 5% CO_2_ Alamar Blue reagent (20 µl, 0.49 mM resazurin in PBS, pH 7.4; Sigma-Aldrich) was added to each well and plates incubated for another 48 hours. For DB829 assays the final cell density was adjusted to 1×10^4^ cells/ml. Alamar Blue was added after 72 hours and incubated for a further 48 hours. Fluorescence (excitation of 530 nm and emission of 590 nm) was measured using a FLUOstar OPTIMA microplate reader (BMG LABTECH) and IC_50_ values determined using GraFit5 (Erithacus Software).

### *In vivo* drug treatments

Mice infected with strain GVR35-LUC2 were treated with trypanocidal compounds after bioluminescence imaging from 21 days post-infection. For diminazene aceturate (Sigma-Aldrich), mice were injected intraperitoneally with a single dose of 40 mg/kg diluted in distilled water. Melarsoprol gel was prepared as described previously [19] and 0.1 ml (containing 3.6 mg melarsoprol) was applied topically to the back of the neck for 3 consecutive days. DB75 [2,5-bis(4-aminidinophenyl)furan] and DB829 (CPD-0801, both provided by Rick Tidwell, University of North Carolina, Chapel Hill) dissolved in DMSO were diluted in distilled water before injecting intraperitoneally into mice. DB75 was injected at 20 mg/kg for 5 consecutive days while four different doses of DB829 were used: 40 mg/kg for 3 days followed by 20 mg/kg for 1 day, 25 mg/kg for 2 days followed by 40 mg/kg for 2 days, 25 mg/kg for 5 days and 20 mg/kg for 4 days.

### Bioluminescence imaging

Prior to bioluminescence imaging, mice were injected with the relevant substrate and imaged under isoflurane anaesthesia using an IVIS spectrum (Caliper Life Science). For LUC2, 150 mg/kg of D-luciferin was injected intraperitoneally 10 minutes prior to imaging while for Rluc, mice were injected intravenously with 15 µg coelenterazine h (Caliper Rediject Coelenterazine h) immediately before imaging. Images were acquired using 10–60 seconds exposure and small or large binning depending on the light produced, 1 f/stop, and an open filter. For whole body images field of view E (25.6×25.6 cm) and for head images field of view A (4×4 cm) was used. Living image software (Caliper Life Sciences) was used for all image acquisition and data analysis. For *ex vivo* imaging of organs mice were perfused using phosphate-buffered saline containing 15 g.L^−1^ glucose. The organs were then removed and soaked in D-luciferin for 5–10 minutes before imaging.

### Multi-photon microscopy

It has been reported that exposure of the dura by local craniotomy impairs brain function [20], [21]. The skull was therefore thinned without removing it, and a two-photon microscope used to image with high resolution through the remaining bone [22]. This technique also enables imaging of objects immediately beneath the skull. Excitation light came from a Ti-sapphire femtosecond laser tunable from 700 to 1050 nm (Chameleon Ultra II, Coherent, Santa Clara, USA). To extend the wavelength range, the output of the Ti-S laser passed through an optical parametric oscillator (OPO, Coherent): when pumped by the Ti-S laser at about 800 nm, outputs up to 1200 nm were obtained. It was possible to use part of the pump wavelength (800 nm) simultaneously with the OPO output. The intensity of the Ti-S beam bypassing the OPO was regulated by an acousto-optical modulator controlled by the imaging program (Zen 2010, Zeiss). The scan head (Zeiss LSM7 MP) had a maximum rate of 8 frames per sec. Almost all the imaging was done with a 20× water immersion objective, NA 1.0. (W Plan-Apochromat, Zeiss). The dichroic mirror in the microscope nose split the beam at a wavelength of 690 nm except when quantum dots emitting at 705 nm were used, in which case a dichroic splitting at 740 nm was used. Five detectors of non-descanned fluorescence were available, three multialkali photodiodes, and two GaAsP detectors. Image files were analysed, and videos prepared, using Volocity (Perkin-Elmer).

### Surgical preparation

Initial anaesthesia of mice was achieved by a low dose of Hypnorm/Hypnovel (VetaPharm/Roche 5 ml/kg body weight, intraperitoneally) and was reinforced as necessary with isoflurane in oxygen. Core temperature was maintained at 36.8–37.0°C by a heating mat (De-Icers (MHG) Ltd, Cheltenham, UK). The parietal skull was exposed and glued to a plate with a hole of 5 mm diameter and the bone within the hole was thinned to about 20 µm [23]. During the thinning and imaging, the skull was superfused with a Tris-buffered saline containing 2 mM CaCl_2_. After imaging, the mouse was humanely killed by an overdose of anaesthetic.

### Exogenous fluorescent labels

Blood plasma was labeled by intravenous injection of dextran 70 kD, 50–70 µl of 100 mg.ml^−1^, conjugated with either fluorescein isothiocyanate or rhodamineB isothiocyanate (both Sigma-Aldrich), or with quantum dots (QTracker, Invitrogen, emission peak at 705 nm, 20–30 µl). For mice infected with WT or LUC2 trypanosomes, a fluorescent diamidine was injected with the vascular marker, usually DB75 [2,5-bis(4-aminidinophenyl)furan] or, occasionally, DB829 (CPD-0801, both provided by Rick Tidwell, University of North Carolina, Chapel Hill) at a final concentration of 10 mg/kg body weight.

### Counting trypanosomes using multi-photon microscopy

Extravascular trypanosomes in the meninges moved too fast to be imaged in three dimensions. However, there was a marked maximum in the population in a layer less than about 10 µm thick. To obtain approximate values for the number of trypanosomes per unit area, areas were chosen at random, and while focused at the depth of maximum trypanosome population, a time series at the maximum scan rate for 100 cycles (giving a total time of 12 s) was acquired. The video was played back at reduced speed and the trypanosomes counted. The size of the imaged area was chosen to include fewer than 20 trypanosomes, and was usually 212 µm^2^ or 143 µm^2^. At least 8 non-overlapping areas were counted in each mouse.

## Results

### Generation of bioluminescent *Trypanosoma brucei brucei*

**Figure 1:**
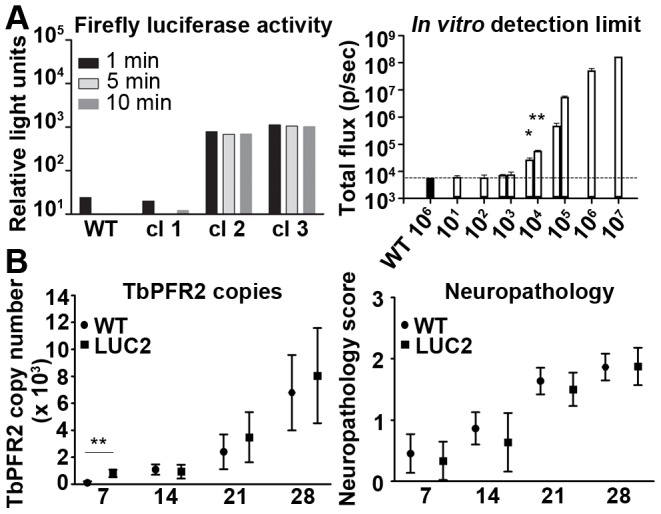
Generation of bioluminescent *T. brucei* GVR35 for detection of trypanosomes *in vivo*. (**A**) Live GVR35 WT and -LUC2 clones were assessed for luciferase activity *in vitro* after addition of D-luciferin. The *in vitro* detection limit was determined by imaging a dilution series of GVR35-LUC2 using IVIS. Data show, on a logarithmic scale, average total flux (in photons per second) ± SD of replicate wells containing a specified trypanosome number. The dotted line indicates background bioluminescence for GVR35 WT. (**B**) Brains of mice infected with WT or GVR35-LUC2 were compared for trypanosome DNA and neuropathology at different times after infection. Real-time qPCR of the trypanosome TbPFR2 gene was performed on brain homogenates. Neuropathology was scored on haematoxylin and eosin stained sections. Graphs show means and 95% confidence interval for each group (*n* = 11–12 per group). *P* value (Student's *t* test) compared to GVR35 WT is indicated *P<0.05; **P<0.01.

To allow *in vivo* tracking of *T. b. brucei* over the full course of infection we generated trypanosomes stably expressing bioluminescent proteins. The optimal reporter vector was determined by employing the monomorphic *T. b. brucei* 427 strain, a well-established laboratory strain that can be genetically modified *in vitro* with ease, and induces a high level of infection in mice within 2–3 days. Bloodstream form 427 trypanosomes transfected with Rluc-pHD309 [15] plasmid, to express Renilla luciferase, or with the TbR-LUC2 (Figure S1), to express firefly luciferase were analyzed *in vitro* and *in vivo* for sensitivity of detection. The majority of 427-Rluc clones exhibited high luciferase activity *in vitro* (∼10^6^ relative light units), similar to the bioluminescent 427 lines generated by Claes *et al* [15]. While 427-LUC2 showed lower luciferase activity *in vitro* (∼10^3^–10^4^ relative light units) these trypanosomes were more readily detected *in vivo* (Figure S2), presumably because the emission wavelength was longer [24]. TbR-LUC2 vector was therefore chosen for generation of the reporter lines to be used in the bioluminescence imaging model. The infection of mice by *T. b. brucei* GVR35 is the standard model for assessing drugs against stage 2 HAT [25], [26]. In order to generate bioluminescent GVR35 trypanosomes the TbR-LUC2 plasmid was transfected into culture-adapted bloodstream form GVR35 parasites. GVR35-LUC2 clone 3 showed the highest luciferase activity (∼10^3^ relative light units) when tested *in vitro* (Figure 1A) and this line was used for further *in vitro* and *in vivo* analyses. To determine the limit of detection, a dilution series of GVR35-LUC2 cells ranging from 10 to 10^7^ trypanosomes were imaged by an *in vivo* imaging system (IVIS) (Figure 1A). Wells containing 10^6^ GVR35 WT trypanosomes were used to determine the background bioluminescence. The minimal trypanosome number reliably detected above background was 5×10^3^ parasites (*P* = 0.015). As seen in Figure 1A, bioluminescence increased with trypanosome number.

### Effect of firefly luciferase expression on GVR35 growth and virulence

A high level of luciferase expression is advantageous for sensitive imaging but might lead to alterations in virulence and disease progression. Bloodstream form GVR35-LUC2 cells were indistinguishable from the GVR35 WT parent line with regards to growth *in vitro* confirming that the pTbR-LUC2 plasmid had no impact on proliferation in culture. To confirm parity between WT and GVR35-LUC2 infections *in vivo*, brains were analysed at 7, 14, 21 and 28 days post-infection for trypanosome load, as assessed by real-time quantitative PCR (qPCR) of parasite DNA [27] and for neuropathology (both described previously in [28] and [29]). No significant difference in parasite burden was detected between WT and GVR35-LUC2 at days 14, 21 or 28 post-infection, although a significant increase in parasite burden was observed at day 7 with GVR35-LUC2 (Figure 1B). Using a well-established neuropathology grading scale [29], WT and GVR35-LUC2 lines produced similar disease scores over the course of the infection. The results suggest that GVR35-LUC2 infections are comparable to WT in respect to brain parasite load and the neuropathological response during the CNS stage of disease.

To ensure that the genetic modification of GVR35 trypanosomes did not influence their sensitivity to trypanocidal compounds the inhibitory concentration (IC_50_) for different trypanocides against WT and GVR35-LUC2 lines was determined using a modified Alamar Blue assay (Table 1). There were no significant differences between WT and GVR-LUC2 in the IC_50_ values for the compounds tested (diminazene, DB75, DB829 and melarsoprol), indicating that LUC2 expression did not influence the sensitivity to these trypanocides *in vitro*.

**Table 1:** *In vitro* sensitivity of GVR35-LUC2 compared to WT for different trypanocidal compounds.

	IC_50_ [nM]†	
Compound	*T. bb* GVR35 WT	*T. bb* GVR35-LUC2
Diminazene aceturate	412.3±9.3	431.2±24.2
DB75	140.6±26.2	140.3±29.5
DB829	525.6±56.5	506.7±50.4
Melarsoprol	15.5±2.4	16.3±3.1

† Data show means ± SD of at least three independent assays with duplicate wells.

### Use of bioluminescent GVR35 to monitor disease

**Figure 2:**
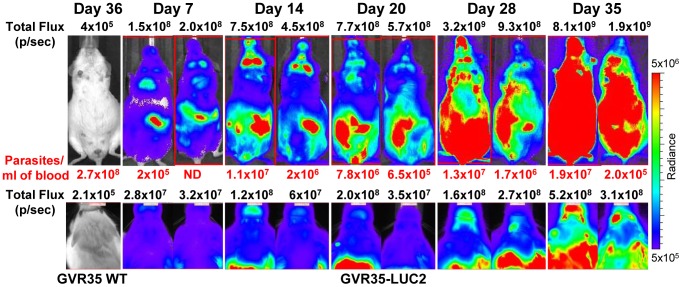
*In vivo* imaging of bioluminescent *T.* **brucei**** **GVR35 over the course of infection.** **** GVR35 WT or -LUC2-infected mice were imaged at different times after infection. Total flux in photons per second (p/sec) for the mouse body (ventral view) or head region (dorsal view) is shown. D-luciferin (150 mg/kg) was injected intraperitoneally 10 minutes before imaging. ND indicates that trypanosomes were not detected in blood samples. Images show the same two representative mice over the full course of infection. Images of a GVR35 WT-infected mouse are shown to indicate the background bioluminescence. The colour scale indicates bioluminescent radiance in photons.second^−1^.centimeter^−2^.steradian^−1^.

**Figure 3:**
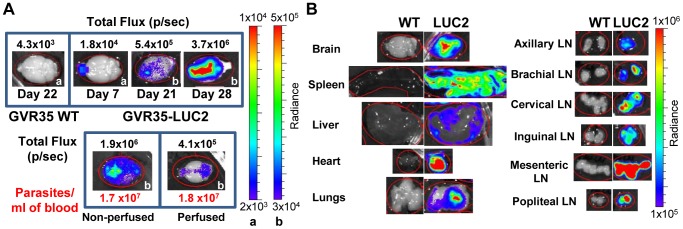
*Ex vivo* imaging of bioluminescent *T.* **brucei**** **GVR35 over the course of infection.** **** (**A**) Brains were removed from perfused GVR35-infected animals at indicated days after infection, soaked in D-luciferin and imaged *ex vivo*. A comparison between brains from perfused and non-perfused animals at day 14 is shown at the bottom. (**B**) Organs were harvested from perfused GVR35 WT or -LUC-infected mice at day 35, soaked in D-luciferin and imaged *ex vivo*. Colour scales indicate bioluminescent radiance. In A, different scales are used for weak (a) and stronger (b) bioluminescent radiance in photons.second^−1^.centimeter^−2^.steradian^−1^.

We next used GVR35-LUC2 to monitor trypanosome burden using IVIS through the full course of an infection. Mice developed fluctuating blood parasitemia [30] and survived up to day 35. Strong bioluminescence was present by day 7 and the signal increased and disseminated to the heads as well as other body regions as the infection progressed (Figure 2). While intermittent decreases in blood parasitemia were observed, bioluminescence increased over time, indicating that the extravascular trypanosome population expands during the course of infection and contributes to the overall bioluminescence. To confirm that GVR35-LUC2 established a CNS infection, brains from infected mice were removed, soaked in D-luciferin and imaged *ex vivo*. Perfusion of mice prior to removal of the brain reduced the bioluminescence by ∼5-fold compared to brains from non-perfused mice, indicating that trypanosomes in the blood serving the brain contribute significantly to total brain bioluminescence (Figure 3A, bottom panel). Therefore, to limit the analysis to extravascular trypanosome loads, mice were routinely perfused before removal of the brains. Imaging over the course of infection revealed the presence of extravascular bioluminescent trypanosomes in brains at day 7 with an increase in bioluminescence, and thus trypanosome loads, over time (Figure 3A, top panel).

We further examined whether this method could be used to detect live trypanosomes in other organs. Mice infected for 35 days were perfused and various organs including brain, spleen, liver, heart, lungs, inguinal-, axilliary-, brachial-, cervical-, mesenteric- and popliteal lymph nodes, were removed and imaged after soaking in D-luciferin (Figure 3B). Bioluminescence was detected in all organs evaluated, indicating the widespread distribution of trypanosomes at the late stage of infection, but also the potential use of this method to evaluate trypanosome burdens in organs during infection.

### Assessment of *in vivo* drug efficacy using the bioluminescent GVR35 model

**Figure 4:**
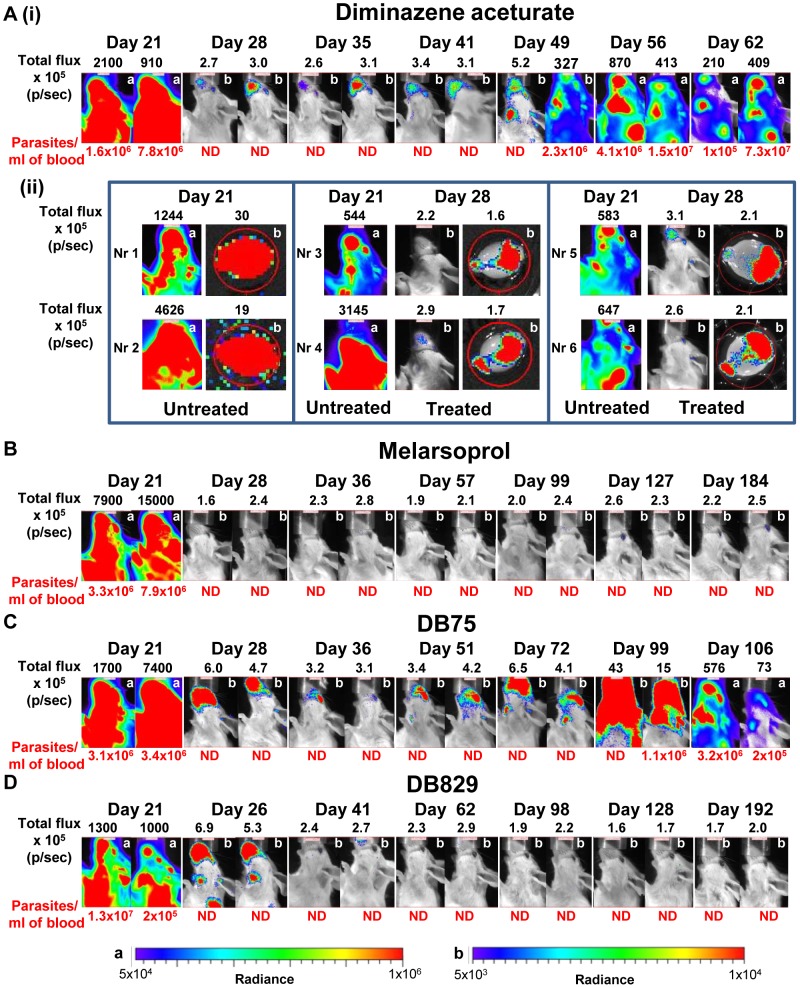
Bioluminescence imaging of *T.* **brucei**** **GVR35-LUC2- infected mice to assess** ***in vivo*** **trypanocidal activity.** **** Mice were treated with (**A**) diminazene aceturate ((i) *n* = 11, (ii) *n* = 6), (**B**) melarsoprol (*n* = 12), (**C**) DB75 (*n* = 6) or (**D**) DB829 (*n* = 8) from day 21 and imaged on indicated days after infection. D-luciferin (150 mg/kg) was injected intraperitoneally 10 minutes before imaging. Bioluminescence from the heads of mice is shown as total flux in photons per second (p/sec). ND indicates that trypanosomes were not detected in blood samples. For each treatment images of the same two representative mice over the entire period are shown. In Aii brains were harvested from perfused GVR35-LUC2-infected mice at day 21 (untreated) or day 35 (diminazene-treated), soaked in luciferin and imaged *ex vivo*. Corresponding untreated heads and treated heads and brains are shown. The same two colour scales are used for all treatments for strong (a) and weaker (b) bioluminescent radiance in photons.second^−1^.centimeter^−2^.steradian^−1^. The colour scale used is indicated in the top right corner of each image.

To validate bioluminescence imaging as a method for evaluating the efficacy of trypanocidal compounds, GVR35-LUC2-infected mice were treated with known trypanocides. We firstly used two well-established drugs: diminazene aceturate (berenil), a first stage drug known to be ineffective for stage 2 [25], and melarsoprol, a drug that clears trypanosomes from the CNS [19], [31]. Mice were imaged at day 21 to confirm that the infection was established, then treated with the relevant compound and imaged weekly. In the first 2 weeks after diminazene treatment most of the bioluminescence detected pre-treatment disappeared and trypanosomes were undetectable in the blood (Figures 4A, S3A and S4A). Head images, however, revealed low bioluminescence, indicative of persistent trypanosomes. Bioluminescence in the heads increased by day 41 with possible localisation in the forebrain and cervical lymph nodes, and spread to the rest of the mouse, and in particular to the spleen in the following weeks. Trypanosomes became detectable in the blood 1–3 weeks after bioluminescence detection by IVIS imaging (Table 2). While bioluminescence was consistently found in the heads of treated mice, the position of the signal varied and often appeared to be located in the rostral area early after treatment. To confirm that trypanosomes did in fact survive in the brain following treatment mice were perfused and brains imaged *ex vivo* (Figure 4Aii). Strong bioluminescence was observed in *in vivo* head and *ex vivo* brain images of untreated mice at day 21 indicating a high level of infection before treatment. Head images of the same mice 1 week after diminazene treatment showed either no signal (mouse nr 3), signals in the rostral area towards the eyes and nose (mouse nr 4, 5, 6) or signals emanating from the inner ear (mouse nr 5 and 6). *Ex vivo* brain images from these mice clearly indicated that bioluminescent trypanosomes were still present in all the brains after treatment, and were predominantly located in the olfactory bulb and cerebellum region.

**Table 2:** *In vivo* antitrypanocidal activity in the GVR35-LUC2 mouse model.

Compound	Dose (days administered)^a^	Relapsed/total	Day p.i. detected by IVIS^b^	Day p.i. detected by microscopy
**Melarsoprol** c	3.6 mg (3)	0/12	None by d184	None by d184
**DB829**	40 mg/kg (3)	0/8^d^	None by d197	None by d197
	20 mg/kg (1)			
**DB829**	25 mg/kg (2)	2/6	d71 (2)	d94 (1), d123 (1)
	40 mg/kg (2)			rest clear until d182
**DB829**	25 mg/kg (5)	1/6^e^	d50 (1)	d77 (1)
				rest clear until d192
**DB829**	20 mg/kg (4)	3/4	d35 (2), d56 (1)	d53 (1)
				all euthanised d60
**DB75**	20 mg/kg (5)	6/6	d36 (3), d57 (1), d64 (1),	d85 (1), d98 (4), d106 (1)
			d85 (1)	
**Diminazene aceturate**	40 mg/kg (1)	11/11	d28 (4), d35 (1), d41 (2),	d49 (4), d56 (5), d62 (2)
			d49 (4)	

a Doses were administered on consecutive days.

b Day post-infection (p.i.) when positive bioluminescence was first detected is shown with number of mice indicated in brackets. The criterion for positive bioluminescence was set at total flux of the head image ≥3×10^5^ photons per second.

c Melarsoprol was applied topically to the back of the neck while the other compounds were injected intraperitoneally.

d 1/8 mice died 4 days after treatment, possibly due to toxicity.

e 1/6 mice was killed on day 44 p.i. due to hind leg paralysis.

Treatment with melarsoprol led to the loss of all bioluminescence within a week (Figures 4B, S3B and S4B) and mice remained aparasitemic and clear of bioluminescence until the end of the experiment at day 184. Quantitative PCR analysis of brains from these mice also showed that they were cleared of trypanosome DNA. Thus the method reliably reported clearance of trypanosomes in cured mice and confirmed previous findings that 3 doses of topical melarsoprol administered during stage 2 disease results in 100% cure rate (Table 2, [19]).

The efficacy of two experimental diamidines was then tested in the GVR35-LUC2 imaging model. DB75 clears trypanosomes from blood but is unable to cure mice during stage 2 disease, while its aza-analog DB829 is effective at curing CNS infections [32], [33]. When GVR35-LUC2-infected mice were treated on day 21 post-infection with the maximum tolerated dose of DB75 (20 mg/kg for 5 days) trypanosomes were cleared from the blood at day 28 but *in vivo* bioluminescence was still detected in the heads and tails (Figures 4C and S3C). By day 36 most of these bioluminescent signals disappeared but remained at a low level in the heads of 50% of treated mice. Bioluminescence reappeared in the heads of the remaining mice over the following weeks (Table 2 and Figure S4C). The bioluminescent signal subsequently increased and spread: at first located in the front of the head, then in the cervical lymph nodes and finally the spleen and rest of body (Figure S3C). Trypanosomes were not detected in the blood until day 85 or later (Figure 4C and Table 2). In DB75-treated mice a bioluminescent signal showing the presence of trypanosomes was detected a full 7 weeks before parasites were first detected in blood.

Dose-dependent *in vivo* activity was observed for DB829 (Table 2 and Figures 4D, S3D, S4D and S5). Treatment with the maximum tolerated dose for this drug (40 mg/kg for 3 days followed by 20 mg/kg for 1 day) resulted in a 100% cure rate. At day 26 trypanosomes could no longer be detected in the blood of treated mice (Figures 4D and S3D) but bioluminescence was still visible in the heads and in the areas of lymph nodes and spleen. Most of this was abolished by day 41 although residual bioluminescent spots (<3×10^5^ photons/sec) were observed in the heads of some mice. In the following weeks mice were cleared of all bioluminescence and no trypanosomes were detected in blood by the endpoints at day 98 or day 197. Lower doses of DB829 were unable to cure all mice: 2/6 relapsed after treatment with 25 mg/kg for 2 days followed by 40 mg/kg for 2 days, 1/6 mice relapsed after treatment with 25 mg/kg for 5 days (Figure S5) and 3/4 relapses occurred at the lowest dose of 20 mg/kg for 4 days (Table 2). A comparison between bioluminescent images of mice that relapsed and those that remained aparasitemic indicated that bioluminescence of 3×10^5^ photons/sec or higher for head regions reliably predicted relapse. This value offers the means to provide a quantitative cut-off with a head signal below 3×10^5^ photons/sec defined as negative. For animals treated with sub-curative doses of DB829 bioluminescence was detected between 3 and 7 weeks before trypanosomes were identified in the blood (Table 2).

### Multi-photon imaging of trypanosome infections in the brain

Trypanosome infection causes meningitis [34], [35] and the presence of trypanosomes in the meninges, and also the superficial parenchyma, has been reported [36]–[38]. If trypanosomes were present in these locations they would contribute to the *in vivo* IVIS signal from the head. Intravital multi-photon microscopy through the thinned skull allowed the identification of individual intravascular and extravascular parasites in the meninges, and below that to a depth of about 150 µm into the parenchyma. The presence of parasite populations in these different regions, and their susceptibility to drug treatments shown by IVIS imaging to be effective against stage 1 or stage 2 disease, could therefore be assessed.

**Figure 5:**
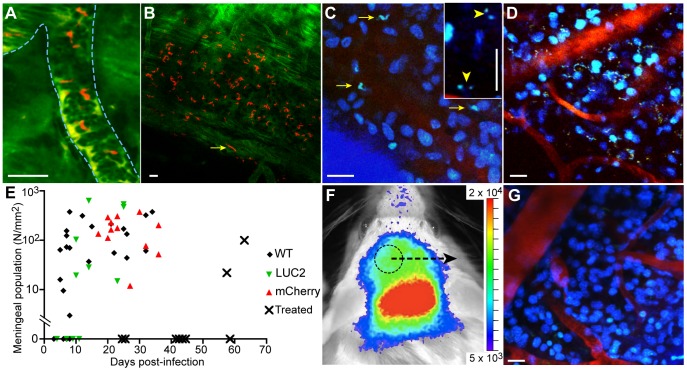
Trypanosomes invade the meninges. (**A**) Lister 427-mCherry trypanosomes (red) in a meningeal blood vessel (outlined by dashed blue lines) at day 3 (Video S1). (**B**) Extravascular GVR35-mCherry trypanosomes (red) day 13. Blood and collagen appear green. A rapidly moving intravascular trypanosome is indicated by the arrow (Video S2). (**C**) 24 hr after intravenous (i.v.) injection of DB75 meningeal trypanosomes are labeled and motile (arrows), as are host nuclei (Video S5), day 28. Inset shows a different mouse at day 10, 25 min after i.v. injection of DB75, only the nucleus and kinetoplast are labeled (arrow heads, Video S3). (**D**) At day 25 numerous GVR35-LUC2 trypanosomes (moving dots in Video S4) are present in the meninges. Labeling by i.v. injection of DB75. (**E**) Numbers of meningeal trypanosomes plotted against days post-infection. Diminazene (40 mg/kg) at day 21 cleared the meninges of trypanosomes until about day 58 (crosses). (**F**) Treatment with diminazene at day 21 reduced luciferase emission but by day 44 there was strong emission from the head, despite zero blood parasitemia. Scale in photons.second^−1^.centimeter^−2^.steradian^−1^. (**G**) Subsequent multi-photon imaging in area circled in (F) showed no motile trypanosomes in the meninges. I.v. injection of DB75 labeled host nuclei. All scale bars 20 µm.

Intravascular trypanosomes were detected through the thinned parietal skull to a depth of about 150 µm below the pia mater. In mice infected for 3 days with *T. b. brucei* strain 427 expressing fluorescent mCherry, trypanosomes were visible in blood vessels, but virtually none were extravascular (Figure 5A and Video S1) demonstrating that the surgery itself did not give trypanosomes access to extravascular spaces. Mice were then infected with *T. b. brucei* strain GVR35 modified to express mCherry and the parasites were imaged between days 13 and 41. Motile GVR35 trypanosomes were observed in extravascular spaces in the meninges. To localize them, we focussed first on the underside of the skull and then imaged deeper into the meninges. The trypanosomes tended to be present in a thin layer, and in each microscope field the depth of greatest trypanosome density was estimated. The mean depth of this peak density was 28.4±6.7 µm (mean ± SD, n = 8 mice) below the skull (Figure 5B and Video S2). To compare results of multi-photon imaging with IVIS imaging it was necessary to use bioluminescent GVR35-LUC2 and WT (as control) trypanosomes. Addition of a fluorescent stain was required to allow detection of trypanosomes using the multi-photon approach and since DB75 accumulates within circulating trypanosomes and has intrinsic fluorescent properties [39] this compound could be used to label trypanosomes. We tested the GVR35 strain and found that, following intravenous injection of DB75 (10 mg/kg), fluorescence-labeled extravascular trypanosomes were clearly visible in the meninges. Within 25 min of DB75 administration, the nucleus and kinetoplast were visible (Figure 5C inset and Video S3), as were nuclei of host cells in the meninges (Figure 5, C and D, and Video S4). Trypanosomes could be unambiguously identified by virtue of their size and motility, which was not affected by DB75 during the maximum of 3 hours imaging per mouse. Furthermore, moving, labeled, trypanosomes were still observed 24 hours after DB75 administration (Figure 5C and Video S5). By comparing the distributions of trypanosomes that expressed fluorescent proteins, and those that were labeled by DB75, we concluded that all the extravascular trypanosomes imaged by multi-photon microscopy were accessible to this compound. Trypanosomes were detected in the meninges from day 5 post-infection with no marked difference in the meningeal population numbers between GVR35 WT, -LUC2 and -mCherry (Figure 5E).

To determine the susceptibility of meningeal trypanosomes to stage 1 drugs, GVR35-infected mice were treated with 40 mg/kg diminazene [25] at day 21. Moving trypanosomes could not be detected in the meninges two days after treatment, indicating that trypanosomes had been cleared from this site (Video S6). Another group of treated mice were imaged on day 44 (23 days after treatment) first by IVIS then by multi-photon imaging. Although no trypanosomes could be detected in the blood of these mice, IVIS imaging revealed bioluminescence in the brain (Figure 5F). However, no trypanosomes were observed by multi-photon microscopy, indicating that IVIS-detected bioluminescence did not originate from meningeal trypanosomes (Figures 5G and S6). At later times, trypanosomes of the various lines used were detected in the meninges but only after their reappearance in the blood (Figure 5E).

## Discussion

Effective treatment of stage 2 HAT requires a drug that crosses the blood-brain barrier in a concentration sufficient to kill CNS-resident trypanosomes. Many trypanocidal drugs, such as those used for stage 1 treatment, clear trypanosomes from the vasculature and peripheral compartment, but parasites that remain viable in the brain will eventually re-establish infection in the blood [11], [25], [40]. Current pre-clinical mouse models of stage 2 HAT assess the efficacy of drugs by the detection of trypanosomes in blood following treatment. In the case of failure, the re-emergence of blood trypanosomes often occurs several months after treatment and the standard model requires 180 days post-treatment follow up to declare a drug curative in stage 2. Here, we describe an infection model that uses bioluminescence imaging to detect trypanosomes in the brain following drug treatment, which is a significant improvement upon the classical 180 day model.

Bioluminescence imaging has been widely used for *in vivo* tracking of tumour cells in cancer and the distribution of pathogens in infected animals [12], [14], [41]–[43]. LUC2, a modified firefly luciferase, has so far provided the best sensitivity for *in vivo* detection [44], [45] and its substrate luciferin is known to cross the blood-brain barrier, allowing successful imaging in the brain [43], . The gene has been optimized for cytosolic expression by the removal of a peroxisomal targeting sequence, which is present at the C-terminal end of the native luciferase gene and shown to target native luciferase to the glycosomes in *T. brucei* [48]–[50]. We found LUC2 firefly luciferase to be superior to Renilla luciferase [15], [16] for *in vivo* detection of *T. b. brucei*, most likely due to its longer wavelength emission (562 nm) [24]. Crucially for the success of the model, the expression of LUC2 from the ribosomal locus in GVR35 remained stable over the course of infection. The TbR-LUC2 plasmid could also be used for the generation of other bioluminescent *Trypanosoma spp.* making it possible to extend the analysis of promising compounds to tests on field isolates.

The GVR35-LUC2 model was validated initially using two widely used trypanocidal drugs: diminazene aceturate, a veterinary drug that can cure stage 1 but not stage 2 disease [25], and melarsoprol, a drug used for stage 2 human disease that cures 100% of treated mice when appropriately dosed [19]. As expected, relapse was observed in the case of diminazene while melarsoprol cured mice, thus providing a benchmark for compounds that cure stage 1 and stage 2 disease. In cases of relapse after treatment with diminazene, bioluminescence was first detected in the heads of mice before disseminating throughout the body. Surprisingly, early signals were often located in rostral areas of the heads in *in vivo* imaged mice, while *ex vivo* brain images clearly showed bioluminescence in the brain. It is feasible that *in vivo* rostral signals are linked to the strong bioluminescence in olfactory bulbs, and the inner ear signals to the cerebellum but this hypothesis requires further investigation. The discrepancy between signal strength detected by *in vivo* and *ex vivo* imaging suggests that much of the brain bioluminescence is lost during *in vivo* imaging, as observed by others [51], or that the substrate D-luciferin does not accumulate in the brain in sufficient amounts with the 150 mg/kg dose used. *In vivo* sensitivity may be improved for future studies with the use of red-shifted luciferases [52], red-shifted luciferin analogues [53] or by increasing the D-luciferin dose [54]. The bioluminescence detected in *ex vivo* brains is consistent with previous observations that trypanosomes survive in the CNS after sub-curative treatment [11], [25] but it does not exclude the possibility that trypanosomes may survive in other extravascular regions. The use of GVR35-LUC2 and *ex vivo* imaging of different tissues early after treatment should be helpful in identifying areas where parasites survive.

For further refinement and testing of the model, groups of mice were treated with two experimental diamidine compounds: DB75, a compound that failed to cure stage 2 models of disease even at the highest tolerated dose, and DB829, shown to have dose-dependent activity against CNS trypanosomes [33]. Importantly, after DB75 treatment, IVIS imaging rapidly revealed that trypanosomes remained viable in the head, even though the blood was cleared. The time taken to identify DB75 as an unsuccessful stage 2 compound was thus reduced by seven weeks using our bioluminescence imaging approach compared to the standard model relying on relapse in the blood.

Although bioluminescence remained visible in some mice within the first 2 weeks after treatment it disappeared in others to re-emerge in the following weeks. To distinguish between cured and non-cured mice it is thus necessary, with the model described here, to extend the imaging period to 60 days post-treatment.

While all bioluminescent signal was cleared one week after melarsoprol treatment, bioluminescence remained visible in mice treated with DB829. This observation may relate to differences in the *in vivo* killing rate and distribution of compounds in extravascular tissues. *In vitro*, melarsoprol kills trypanosomes more rapidly than DB829 [55]–[58]. In addition to providing information about relapse, the *in vivo* imaging model applied here can therefore distinguish *in vivo* between fast-acting compounds, which may be more favourable, and those with slower activity.

Despite having a higher IC_50_ value than DB75 in *in vitro* testing (Table 1, [33], [58]), DB829 was more active against CNS trypanosomes *in vivo*. This discrepancy between *in vitro* and *in vivo* efficacy may be explained by different distribution of these two compounds *in vivo*. Studies using the prodrug of each compound have shown higher systemic levels of DB829 compared to DB75 [59] but it is not known whether this accounts for its greater efficacy in the brain. Differences in the uptake of diamidines by trypanosomes grown *in vitro* and *in vivo* have also been described [58]. Such observations highlight the need for *in vivo* assessment of compounds under development as stage 2 drugs, and extension of pharmacokinetic studies to assess distribution of drugs in the CNS and their uptake by resident trypanosomes.

Using multi-photon imaging, which provides optical resolution of individual parasites, we observed invasion of the meninges as early as day 5 post-infection [37], [38]. These parasites are likely to be responsible for some of the bioluminescent signal emanating from the head. However, clearance by diminazene indicates that this population is not protected by the blood-brain barrier, and does not significantly contribute to bioluminescent signals emanating from the head following chemotherapy. The meningeal trypanosomes are thus not responsible for the later relapse observed in mice treated with stage 1 drugs. These findings extend our understanding of CNS-associated trypanosomes to include populations that enter the CNS during earlier stages of the infection and are accessible to stage 1 drugs.

Trypanosomes in the superficial meninges of mice may be equivalent to the so-called ‘intermediate stage’ of infection in some patients with HAT. This intermediate stage of infection has been suggested following successful treatment of patients presenting with raised CSF white blood cell counts with stage 1 drugs [60]–[62], though the concept and treatment of this clinical scenario remains controversial. The multi-photon imaging approach used in this study revealed a location in the brain where trypanosomes are accessible to stage 1 drugs, but we were unable to identify parasites within parenchyma beyond the blood-brain barrier, protected from stage 1 treatments, that were responsible for the eventual recrudescence of parasitaemia. Further investigations, utilising this powerful technique, will provide invaluable information about trypanosome interactions and distribution throughout the brain. For example with the use of fine optical probes such as gradient index (GRIN) lenses the range of multiphoton imaging could be extended to reach several millimetres into the brain [63], [64].

In summary, we have developed *in vivo* bioluminescence imaging to extend the capability of the currently employed *T. brucei* strain GVR35 model of stage 2 disease. The model provides a substantial improvement in time taken to establish whether a compound is curative in stage 2 disease. The time taken to assess drug efficacy in stage 2 trypanosomiasis has been a major bottle neck in the drug development process and the profound reduction in screening time we present here, cutting the post-treatment follow up time by two thirds, represents a significant advance that should expedite drug discovery for stage 2 HAT.

## Supporting Information

Figure S1 **Vector map of the TbR-LUC2 plasmid.** Expression of firefly luciferase (LUC2) is under the control of an *rDNA* promoter with a *GPEET* 5′UTR and *αβ Tubulin* 3′UTR. The plasmid contains a puromycin resistance cassette with a *αβ Tubulin* 3′UTR to allow for the antibiotic selection of trypanosomes that integrate the construct. An *rDNA* spacer was added at the 3′end enabling integration of *SacI*-*KpnI* linearized plasmid into the *rDNA* loci of trypanosomes. The size of each DNA fragment in kilobase (kb) is indicated as well as the restriction sites used for cloning.
(TIFF)

Figure S2 **Generation of bioluminescent** ***T. brucei*** **427 to determine the optimal reporter for** ***in vivo*** **imaging.** (**A**) Live *T. b.* 427 expressing Rluc or LUC2 were assessed for luciferase activity *in vitro* after addition of appropriate substrates. (**B**) Mice infected with 427 WT, 427-Rluc (clone 3) or 427-LUC2 (clone 4) were imaged by IVIS after intraperitoneal administration of D-luciferin (LUC2) or intravenous administration of coelenterazine (RLuc). Total flux in photons per second (p/sec) shows bioluminescence over the mouse body. ND indicates that trypanosomes were not detected in blood samples. Representative images of individual mice within each group are shown (*n* = 3). The colour scale indicates bioluminescent radiance in photons.second^−1^.centimeter^−2^.steradian^−1^.
(TIFF)

Figure S3 **Bioluminescence imaging of GVR35-LUC2-infected mice to assess** ***in vivo*** **trypanocidal activity.** Mice were treated with (**A**) diminazene aceturate (*n* = 11), (**B**) melarsoprol (*n* = 12), (**C**) DB75 (*n* = 6) or (**D**) DB829 (*n* = 8) from day 21 post-infection (see Materials and Methods for dosing regimens) and imaged weekly after drug administration. Bioluminescence from the bodies or heads of infected mice following injection of D-luciferin (150 mg/kg) is shown as total flux in photons per second (p/sec). ND indicates that trypanosomes were not detected in blood samples. For each treatment images of the same two representative mice over the entire period are shown. The same two colour scales are used for all treatments to indicate strong (a) and weaker (b) bioluminescent radiance in photons.second^−1^.centimeter^−2^.steradian^−1^. The colour scale used is indicated in the top right corner of each image.
(TIFF)

Figure S4 **Bioluminescence over time in GVR35-LUC2-infected mice to assess** ***in vivo*** **trypanocidal activity.** Mice were treated with (**A**) diminazene aceturate (*n* = 11), (**B**) melarsoprol (*n* = 12), (**C**) DB75 (*n* = 6) or (**D**) DB829 (*n* = 8) from day 21 post-infection (see Materials and Methods for dosing regimens) and imaged weekly after drug administration. Plots show total flux in photons per second for whole bodies (left) and heads (right) of all mice in each treatment group. The time of the bioluminescence measurement in days post-infection is shown on the x-axis.
(TIFF)

Figure S5 **Bioluminescence imaging of GVR35-LUC2-infected mice to assess** ***in vivo*** **trypanocidal activity of DB829.** Mice were treated with 25 mg/kg of DB829 for 5 consecutive days from day 21 post-infection (p.i.) and imaged weekly after injection of D-luciferin (150 mg/kg). Bioluminescence from the bodies or heads of infected mice is shown as total flux in photons per second (p/sec). ND indicates that trypanosomes were not detected in blood samples. Images of the same two representative mice over the entire period are shown. For day 113 and 192 p.i. only one mouse is shown because the relapsed mouse was euthanised. Different scales are used for strong (a) and weaker (b) bioluminescent radiance in photons.second^−1^.centimeter^−2^.steradian^−1^.
(TIFF)

Figure S6 **Trypanosomes in the meninges contribute little to the IVIS signal.** The mean luciferase radiance from an area over the parietal skull that approximated the area subsequently imaged by two-photon microscopy was measured (see Figure 5E). Trypanosomes were rendered fluorescent by i.v. injection of DB75 and imaged through the thinned skull (as for Video S3). Approximate mean numbers per unit area of meninges were calculated for each mouse by counting extravascular trypanosomes in videos from 8–10 randomly selected fields of view. In each field, the imaging plane was set so that the greatest number of trypanosomes was observed, and all trypanosomes that appeared during 12 s of imaging were counted. This method will have systematic errors, but, in general, the trypanosomes were confined to a range of depths sufficiently small that during the 12 s of imaging nearly all the trypanosomes moved into the imaging plane and were detected.
(TIFF)

Table S1 **Primer sequences used to amplify the** ***rDNA*** **promoter, spacer, UTR fragments and reporter genes (the incorporated restriction sites are underlined).**
(DOC)

Video S1 **Intravascular** ***T. brucei*** **427-mCherry trypanosomes.** 3 days p.i. Blood marker dextran-fluorescein, 70 kD. Real time. Excitation wavelength 1050 nm. The width of the frame is 68 microns.
(MOV)

Video S2 **Extravascular** ***T. brucei*** **GVR35-mCherry trypanosomes in the superficial meninges.** 13 days p.i. Collagen fibres of the dura are revealed by second harmonic generation (green). Approximate real time. Width of frame 424 microns.
(MOV)

Video S3 **Intravascular DB75 (10 mg/kg) labels nuclei and kinetoplasts of extravascular trypanosomes in the meninges.** Excitation wavelength 750 nm. Width of frame 121 microns.
(MOV)

Video S4 **Luciferase-expressing trypanosomes at 25 days p.i.** Trypanosomes fluorescence labeled by i.v. injection of DB75. Width of frame 424 microns.
(MOV)

Video S5 **DB75 does not abolish movement of extravascular trypanosomes 24 h after i.v. injection.** Z projection of a stack 5 microns deep. Occasional trypanosomes appear with emission from the whole body extending into the green, and there are stationary green objects which may be debris. Excitation wavelength 800 nm. Blood marker dextran-rhodamine. Width of frame 105 microns.
(MOV)

Video S6 **A companion mouse to that of Video S5 was treated with diminazene (40 mg/kg) at 21 days p.i. and imaged at 44 days p.i.** No trypanosomes are detected as moving spots. Approximate real time. Width of frame 424 microns.
(MOV)
