# Supplementary figures and images for: In Vivo Imaging of Trypanosome-Brain Interactions and Development of a Rapid Screening Test for Drugs against CNS Stage Trypanosomiasis
Source: PLoS Negl Trop Dis. 2013 Aug 22;7(8):e2384. doi: 10.1371/journal.pntd.0002384 (PMC3749981; doi:10.1371/journal.pntd.0002384)

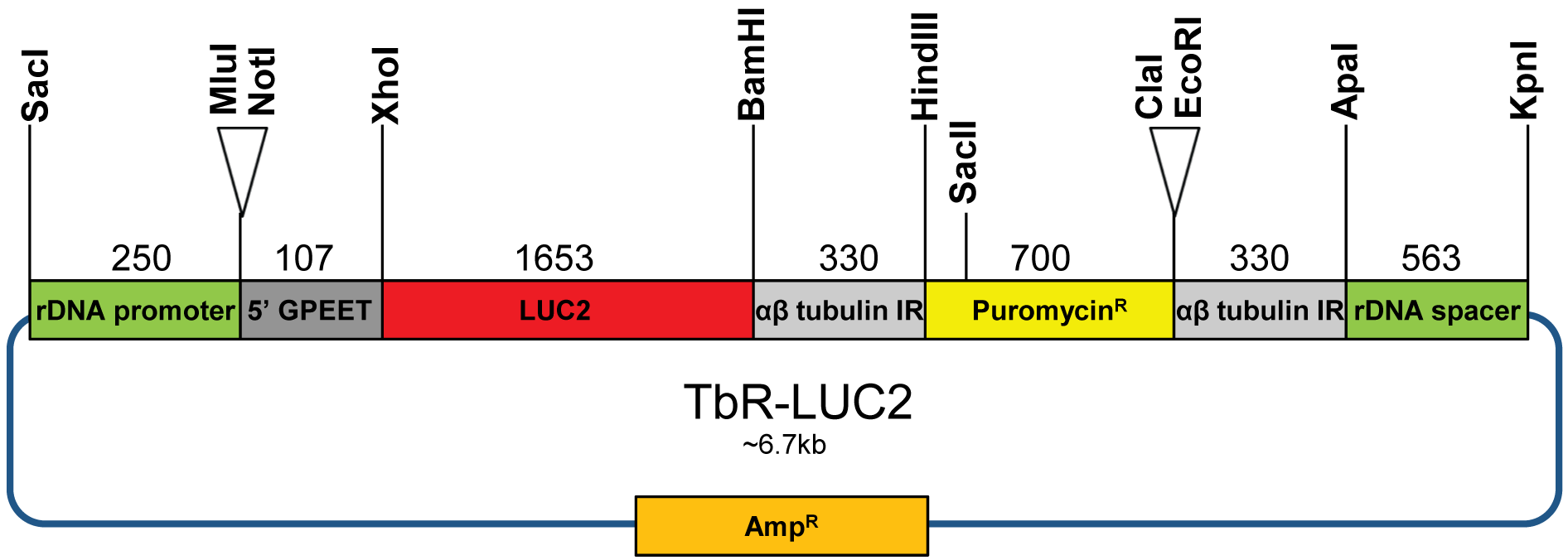

Supplement: Figure S1 — Vector map of the TbR-LUC2 plasmid. Expression of firefly luciferase (LUC2) is under the control of an rDNA promoter with a GPEET 5′UTR and αβ Tubulin 3′UTR. The plasmid contains a puromycin resistance cassette with a αβ Tubulin 3′UTR to allow for the antibiotic selection of trypanosomes that integrate the construct. An rDNA spacer was added at the 3′end enabling integration of SacI-KpnI linearized plasmid into the rDNA loci of trypanosomes. The size of each DNA fragment in kilobase (kb) is indicated as well as the restriction sites used for cloning. (TIFF) [file pntd.0002384.s001.tiff]

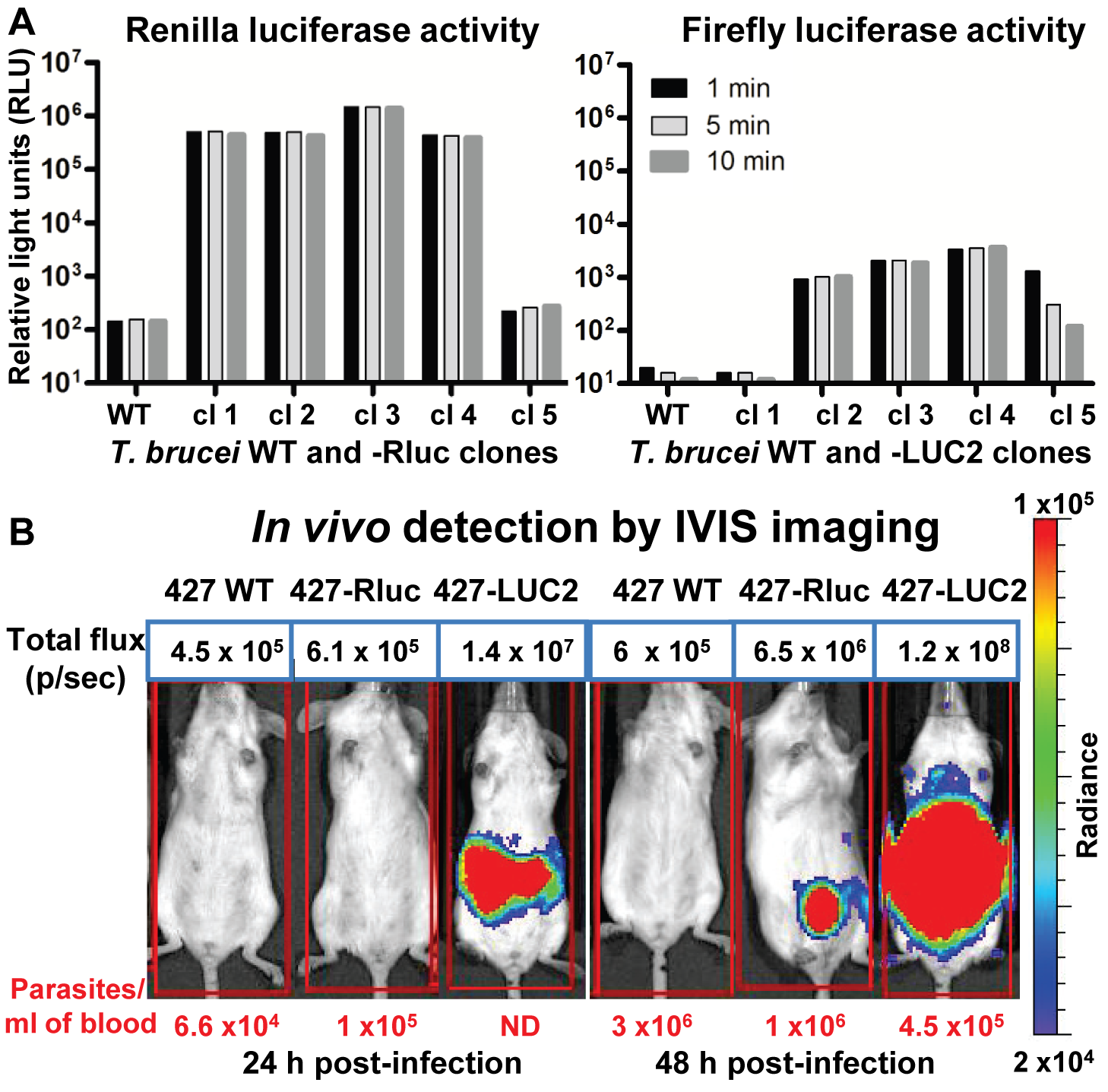

Supplement: Figure S2 — Generation of bioluminescent T. brucei 427 to determine the optimal reporter for in vivo imaging. (A) Live T. b. 427 expressing Rluc or LUC2 were assessed for luciferase activity in vitro after addition of appropriate substrates. (B) Mice infected with 427 WT, 427-Rluc (clone 3) or 427-LUC2 (clone 4) were imaged by IVIS after intraperitoneal administration of D-luciferin (LUC2) or intravenous administration of coelenterazine (RLuc). Total flux in photons per second (p/sec) shows bioluminescence over the mouse body. ND indicates that trypanosomes were not detected in blood samples. Representative images of individual mice within each group are shown (n = 3). The colour scale indicates bioluminescent radiance in photons.second−1.centimeter−2.steradian−1. (TIFF) [file pntd.0002384.s002.tiff]

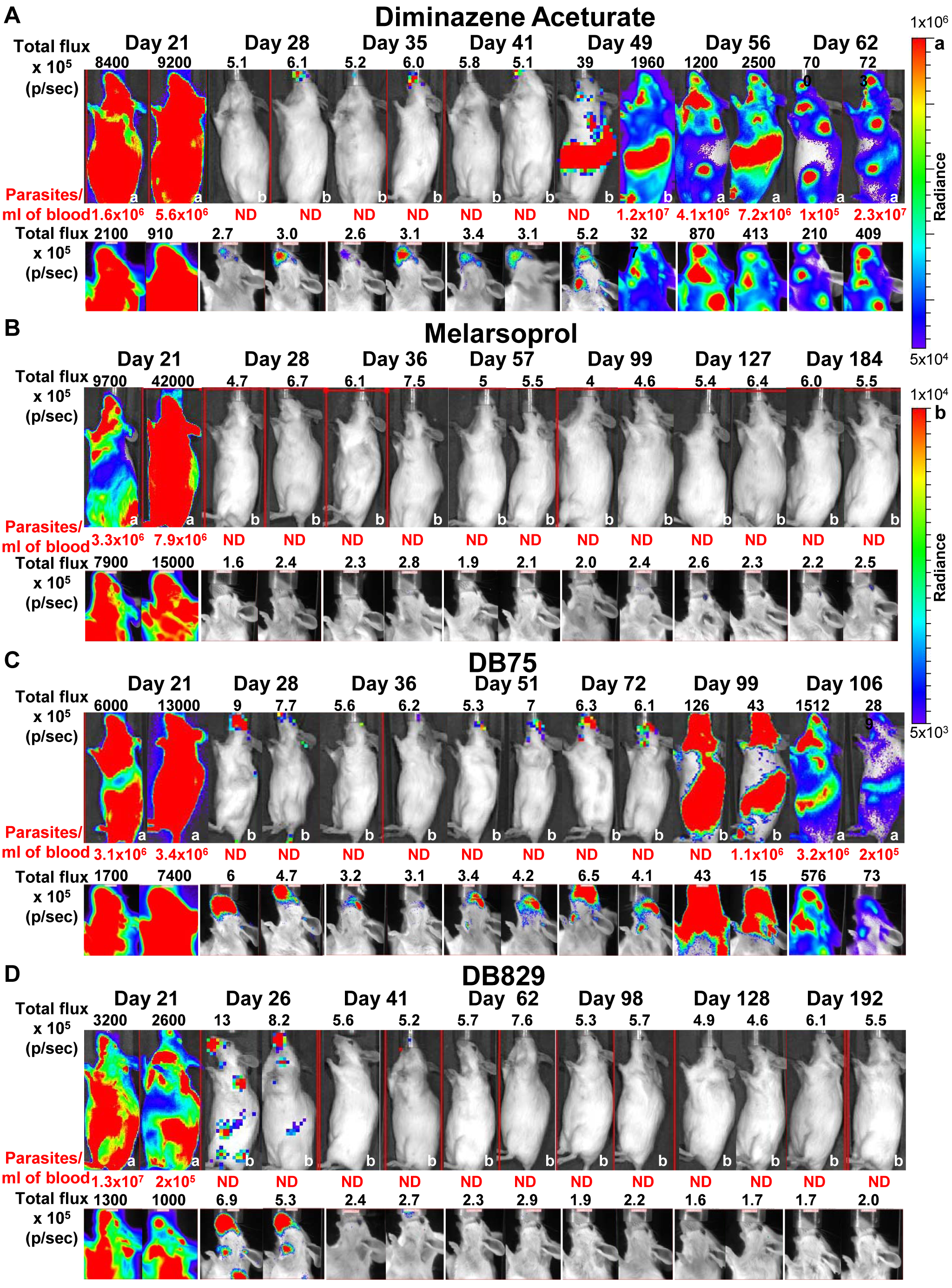

Supplement: Figure S3 — Bioluminescence imaging of GVR35-LUC2-infected mice to assess in vivo trypanocidal activity. Mice were treated with (A) diminazene aceturate (n = 11), (B) melarsoprol (n = 12), (C) DB75 (n = 6) or (D) DB829 (n = 8) from day 21 post-infection (see Materials and Methods for dosing regimens) and imaged weekly after drug administration. Bioluminescence from the bodies or heads of infected mice following injection of D-luciferin (150 mg/kg) is shown as total flux in photons per second (p/sec). ND indicates that trypanosomes were not detected in blood samples. For each treatment images of the same two representative mice over the entire period are shown. The same two colour scales are used for all treatments to indicate strong (a) and weaker (b) bioluminescent radiance in photons.second−1.centimeter−2.steradian−1. The colour scale used is indicated in the top right corner of each image. (TIFF) [file pntd.0002384.s003.tiff]

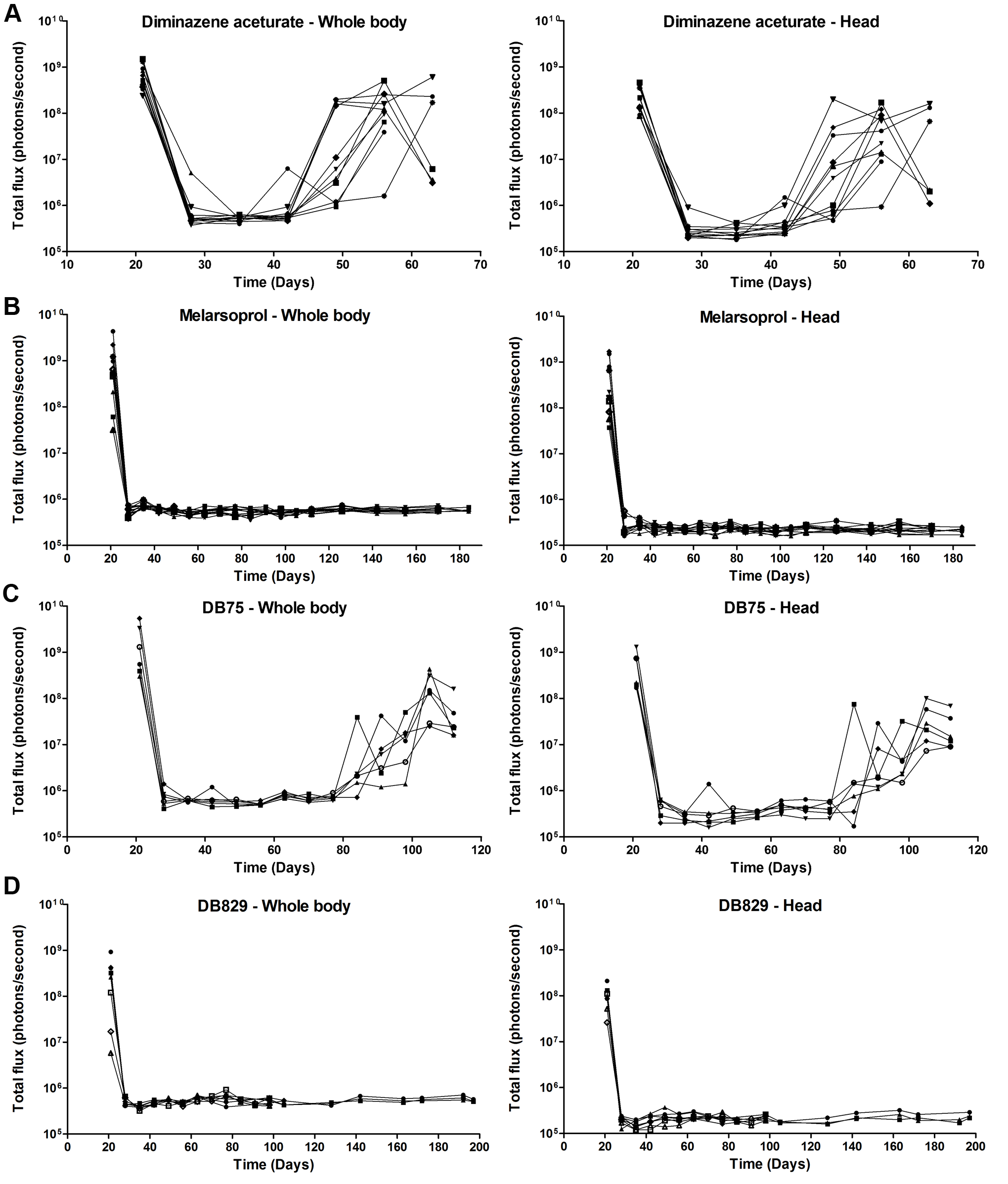

Supplement: Figure S4 — Bioluminescence over time in GVR35-LUC2-infected mice to assess in vivo trypanocidal activity. Mice were treated with (A) diminazene aceturate (n = 11), (B) melarsoprol (n = 12), (C) DB75 (n = 6) or (D) DB829 (n = 8) from day 21 post-infection (see Materials and Methods for dosing regimens) and imaged weekly after drug administration. Plots show total flux in photons per second for whole bodies (left) and heads (right) of all mice in each treatment group. The time of the bioluminescence measurement in days post-infection is shown on the x-axis. (TIFF) [file pntd.0002384.s004.tiff]

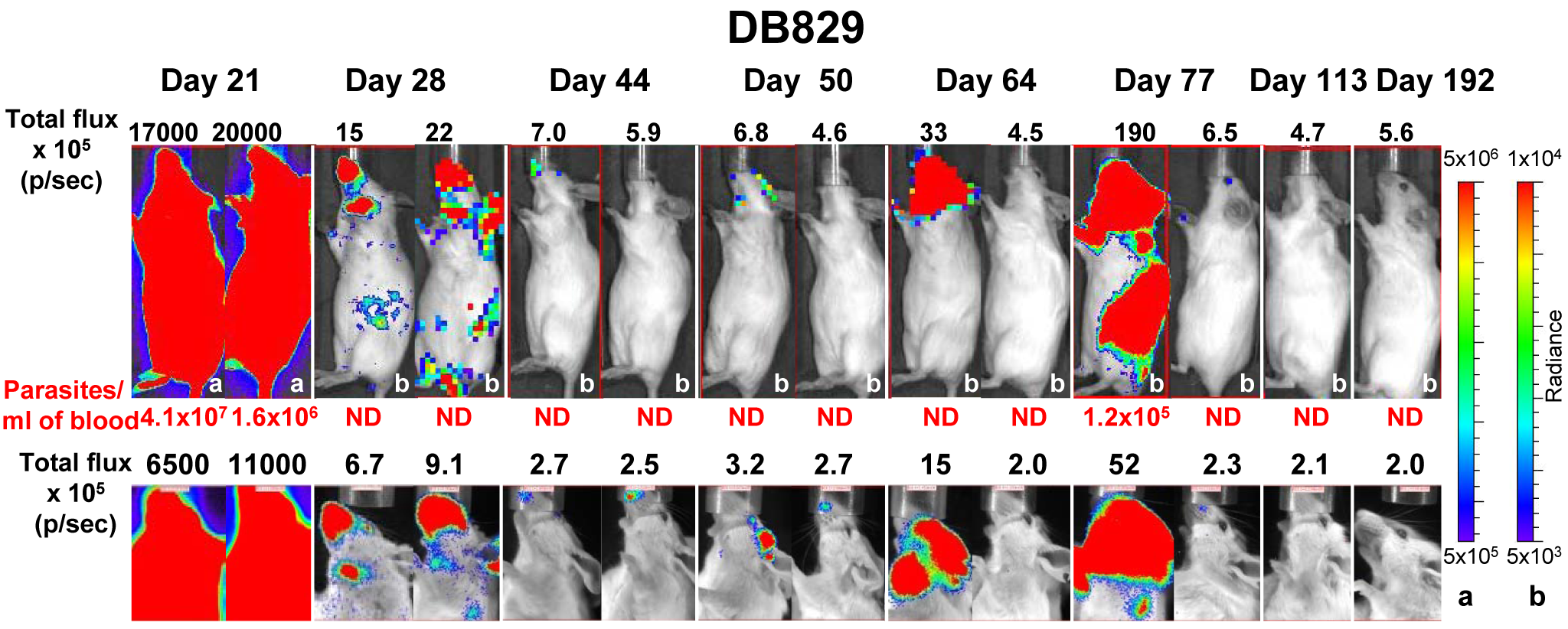

Supplement: Figure S5 — Bioluminescence imaging of GVR35-LUC2-infected mice to assess in vivo trypanocidal activity of DB829. Mice were treated with 25 mg/kg of DB829 for 5 consecutive days from day 21 post-infection (p.i.) and imaged weekly after injection of D-luciferin (150 mg/kg). Bioluminescence from the bodies or heads of infected mice is shown as total flux in photons per second (p/sec). ND indicates that trypanosomes were not detected in blood samples. Images of the same two representative mice over the entire period are shown. For day 113 and 192 p.i. only one mouse is shown because the relapsed mouse was euthanised. Different scales are used for strong (a) and weaker (b) bioluminescent radiance in photons.second−1.centimeter−2.steradian−1. (TIFF) [file pntd.0002384.s005.tiff]

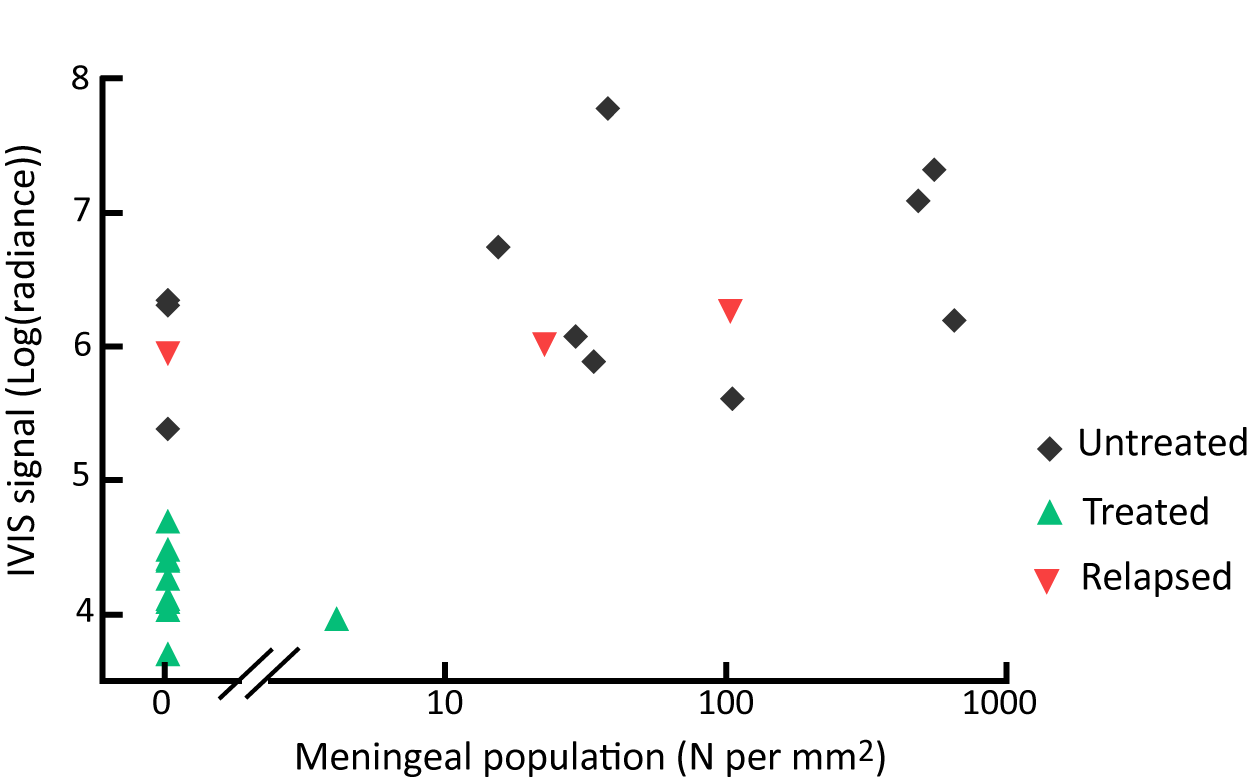

Supplement: Figure S6 — Trypanosomes in the meninges contribute little to the IVIS signal. The mean luciferase radiance from an area over the parietal skull that approximated the area subsequently imaged by two-photon microscopy was measured (see Figure 5E). Trypanosomes were rendered fluorescent by i.v. injection of DB75 and imaged through the thinned skull (as for Video S3). Approximate mean numbers per unit area of meninges were calculated for each mouse by counting extravascular trypanosomes in videos from 8–10 randomly selected fields of view. In each field, the imaging plane was set so that the greatest number of trypanosomes was observed, and all trypanosomes that appeared during 12 s of imaging were counted. This method will have systematic errors, but, in general, the trypanosomes were confined to a range of depths sufficiently small that during the 12 s of imaging nearly all the trypanosomes moved into the imaging plane and were detected. (TIFF) [file pntd.0002384.s006.tiff]
