# Supplementary material for: In Vivo Imaging of Trypanosome-Brain Interactions and Development of a Rapid Screening Test for Drugs against CNS Stage Trypanosomiasis
Source: PLoS Negl Trop Dis. 2013 Aug 22;7(8):e2384. doi: 10.1371/journal.pntd.0002384 (PMC3749981; doi:10.1371/journal.pntd.0002384)
Supplement: Table S1 — Primer sequences used to amplify the rDNA promoter, spacer, UTR fragments and reporter genes (the incorporated restriction sites are underlined). (DOC) [file pntd.0002384.s007.doc]

**Table S1: Primer sequences used to amplify the *rDNA* promoter, spacer, UTR fragments and reporter genes (the incorporated restriction sites are underlined).**

| **Primer sets** | | **Sequence** |
| --- | --- | --- |
| ***rDNA 5’ promoter*** | | |
| *Fwd* | *SacI* | *GAGCTCCCTGCAGGCTTTCCACCCAGCGCGGGTGCATTCT* |
| *Rev* | *MluI/NotI* | *ttttgcggccgcacgcgtGTACGCCGTAAGCGCTACTTTTACTGC* |
| ***rDNA 3’ spacer*** | | |
| *Fwd* | *ApaI* | *aaaagggcccGAATTCGATGCGAGGCGAATCGCTCAG* |
| *Rev* | *KpnI* | *ttttggtaccGAATTCGAGCTCGGCGCGCCATATAGT* |
| ***GPEET 5’ UTR*** | | |
| *Fwd* | *NotI* | *GCGGCCGCCTGCACGCGCCTTCGAGTTTTTTTTCCTTTTCCCCATTTTTTTCAACTTGAAGACTTC* |
| *Rev* | *XhoI* | *CTCGAGGTGAATTTTACTTTTTGGTGTAATTGAAGTCTTCAAGTT* |
| ***luc2 gene*** | | |
| *Fwd* | *XhoI* | *GCACTCGAGatggaagatgccaaaaacattaagaagggcc* |
| *Rev* | *BamHI* | *TGCGGATCCTCAATGATGATGATGATGATGCACGGCGATCTTGCCGCCCTTCTTGGCC* |
| ***mCherry gene*** | | |
| *Fwd* | *HindIII* | *CGCAAAGCTTATGGTTAGTAAAGGAGAAGAAAATAACATGGC* |
| *Rev* | *BamHI* | *TGCGGATCCTCAATGATGATGATGATGATGTTTGTATAGTTCATCCATGCCAC* |
